# Feasibility of Virtual Assessment of Physical Frailty in Solid Organ Transplant Recipients: A Single Center, Observational Study

**DOI:** 10.5195/ijt.2022.6447

**Published:** 2022-06-03

**Authors:** Manoela de Paula Ferreira, Noori Chowdhury, Lisa Wickerson, Heather Ross, Nazia Selzner, S. Joseph Kim, Lianne G. Singer, Sunita Mathur

**Affiliations:** 1 Department of Physical Therapy, University of Toronto, Toronto, Ontario, Canada; 2 Ajmera Transplant Centre, University Health Network, Toronto, Ontario, Canada; 3 Medicine, Nephrology, University Health Network, Toronto, Ontario, Canada; 4 Medicine, Respirology, University Health Network, Toronto, Ontario, Canada; 5 School of Rehabilitation Therapy, Queen’s University, Kingston, Ontario, Canada.

**Keywords:** COVID-19, Frailty, Telehealth, Transplantation

## Abstract

**Objectives:**

To describe the feasibility of virtual assessments of physical frailty in solid organ transplant (SOT) recipients using a modified Fried Frailty Index (mFFI) and Short Physical Performance Battery (SPPB), and to describe the prevalence of frailty 12-months post-transplant using virtual assessment.

**Methods:**

Virtual assessments were performed using an e-questionnaire and a video-call for functional tests. Feasibility variables included: internet quality, video-call duration, presence of a companion, and adverse events.

**Results:**

34 SOT recipients, median age 62 (46-67), 76% lung recipients, 47% female, were included. The video-call had a median duration of 12 minutes (10-15 min), without adverse events. A companion was present in 23 (68%) video-call assessments. Fifteen SOT recipients (44%) were classified as pre-frail by the mFFI, and none were frail. Three participants (8.8%) were classified as frail using the SPPB.

**Conclusion:**

Virtual frailty assessments can be used as an alternative to in-person assessments in SOT recipients.

Frailty is an increased state of vulnerability due to a decline in function across multiple physiologic systems (Exterkate et al., 2016; Watford et al., 2021). Among solid organ transplant (SOT) patients, frailty is an important clinical marker associated with increased risk of waitlist death/delisting, post-transplant mortality in kidney, heart, and lung transplant recipients (Montgomery et al., 2020; Varughese at al., 2020, 2021), and increased risk of graft dysfunction and delirium in kidney transplant recipients (Harhay et al., 2020). The most common measure of physical frailty is the Fried Frailty Index (FFI) (Fried et al., 2001), which includes five domains: muscle weakness, low physical activity, weight loss, slowness, and exhaustion (Kobashigawa et al., 2019). Recently, the Short Physical Performance Battery (SPPB), which includes an assessment of gait speed, balance, and lower body strength, has also been used as an alternative option to measure physical frailty in adult lung (Singer et al., 2018; Wickerson et al., 2020) and kidney (Schaenman et al., 2019) transplant candidates. Both physical frailty measures require the participant to perform functional tests that typically are done through in-person clinical assessment.

The COVID-19 pandemic has posed limitations regarding in-person contact for all types of activities, including hospital or clinic visits. These restrictions increased the need for telehealth (Portnoy et al., 2020) to minimize the risk of cross-infection, especially in older people with chronic diseases or immunocompromised individuals, such as SOT recipients. It is expected that telehealth services will continue beyond the pandemic (Galea, 2019). Thus, there is a need to understand how frailty assessments can be done using virtual methods.

Compared with an in-person assessment of physical frailty, there are fewer studies on conducting functional tests for physical frailty using telehealth. In a study by Park et al. (2021), sensors placed on the lower limb joints were used to derive three components of the FFI, weakness, slowness, and exhaustion, using only the 5-times sit to stand test. Zahiri et al. (2020) used kinematic elbow flexion/extension features determined from video analysis to remotely evaluate frailty (slowness, weakness) in people with COPD. Although these studies developed new ways to assess physical frailty remotely, they did not aim to report on the feasibility of performing virtual frailty assessments, including functional tests, or use the standard clinical protocols for conducting these tests.

A recent study in liver transplant recipients (Keating et al., 2020) investigating the agreement and reliability between in-person measures performed by a clinician with participants' self-measures done virtually found that in-person and virtual assessments were reliable. The study assessed clinical data (body weight, systolic and diastolic blood pressure, and waist circumference) and functional assessments (repeated chair sit-to-stand, maximal push-ups, and the 6-minute walk test). However, there was wide individual variability in accuracy and agreement, with none of the functional assessments being performed within acceptable limits relative to minimal clinically important differences.

The objectives of this study were to (1) describe the feasibility of conducting a virtual assessment of physical frailty in SOT recipients using a modified FFI and SPPB, and (2) describe the prevalence of physical frailty in SOT recipients at 12 months post-transplant using these measures.

## METHODS

### STUDY DESIGN

This is an implementation feasibility study (Bowen et al., 2009) with secondary analysis of a larger prospective, single-center cohort study - Frailty and Sarcopenia in Organ Transplantation (FROST), conducted at the Ajmera Transplant Centre, University Health Network. The study was approved by the University Health Network Research Ethics Board (REB # 18-5428) and the University of Toronto Health Sciences REB (REB # 36493). Participants provided written, informed consent before undergoing study procedures. The study was planned for in-person study visits. However, the virtual assessment was developed in response to COVID-19 restrictions placed in March 2020, when participants were no longer allowed to come to the hospital solely for research study visits. For this part of the study, an amendment consent was provided over the phone between the participant and the study coordinator.

Participants were adults (18 years and over), who had received a solid organ transplant (lung, liver, heart, kidney), who were tested using virtual assessment at the 12-month post-transplant timepoint and had performed the SPPB test in an in-person FROST study session pre-transplant were included. Participants who could not understand the study questionnaires, follow instructions, or did not have access to an internet connection were excluded from the study.

### PROCEDURES

**Figure 1:**
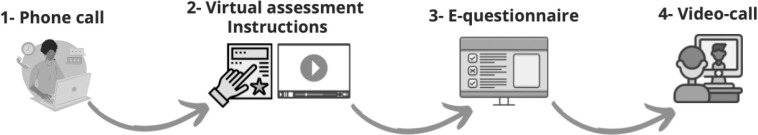
Components of the Virtual Assessment

The virtual assessment consisted of four components, as shown in Figure 1. First, a phone call was made to the participant to explain the reason for the shift from an in-person study visit to a virtual assessment and to determine if the participant had an internet connection and smartphone, tablet, or laptop for the video-call. Second, an email was sent with the virtual assessment instructions. This included a video demonstration of the functional tests, developed by the investigators for the study (https://youtu.be/N_lqTuj5A2o), the Microsoft Teams link for the video-call and a list of the space/equipment requirements for the functional tests: chair with back support and no armrests (e.g., dining chair), measuring tape, 5 × 3 meters flat, open space.

Third, an electronic questionnaire was sent by email with three FFI components: (1) Exhaustion was measured by two epidemiologic studies' depression questions (CES-D) (Bernabeu-Mara et al., 2015); (2) The shrinking measurement was obtained by the self-report weight loss of at least ten pounds in the last year (Abellan van Kan et al., 2009); and finally, (3) The Duke Activity Status Index (DASI) questionnaire measured low activity using sex-based cut-offs (Hltaky et al., 1989). The DASI questionnaire was an alternative to the Minnesota Leisure Time Physical Activity (MLTA) questionnaire, as DASI seems to be more accurate to measure physical frailty in adults with advanced lung disease or critical illness (Baldwin et al., 2017).

The modified FFI (mFFI) score combined the e-questionnaire and the video-call. Hence, as a fourth step of the virtual assessment, the study investigator (MF) made a video-call to the participant. First, information related to weight and height was reported by the participant. After that, the fourth FFI component (modified weakness) was assessed by the question: Do you have difficulty opening a jar of jam (or jar of something else) that has never been opened? (Simard et al., 2012) where ‗yes‘ was recorded as the presence of weakness. This question replaced the handgrip strength test, traditionally used to measure the FFI weakness. The fifth FFI component (slowness) was measured by the highest SPPB gait speed, stratified by sex and height. The FFI total score was used to stratify individuals based on their score: non-frail = 0, pre-frail = 1-2, frail = 3-5 (Fried et al., 2001).

All components of the SPPB were observed and measured through the video-call: for the three measures of static standing balance participants were instructed to be near a wall for support in case of unsteadiness, gait speed over 5 meters, and 5-times to sit-to-stand test (having the chair set-up against the wall). For gait speed, participants were asked to measure the floor in a straight distance of 5 meters and mark the floor at the start (0-meter mark), and at 4 meters and 5 meters from the starting point, by placing a visible marker (e.g., a shoe) at each point. The assessor verbally checked that the participant had placed the markers as instructed. Gait speed was measured over the first 4 meters, with the last meter being a deceleration path. The performance on each component is scored out of 4 (maximal score of 12), with higher scores indicating increased functional mobility. Gait speed and 5-times sit-to-stand test were performed twice, and the highest score for each one was included in the total score. For SPPB, a total score < 9 was used as the cut-off for physical frailty (Guarini et al., 1994). Timing of the SPPB components was done by the same study investigator using a stopwatch.

At the end of the video-call, the assessment duration, internet connection (speed delays or gaps), presence of companion (yes/no), and adverse events (e.g., falls, dyspnea or pain) were documented for feasibility purposes. The participants' responses were also recorded for the following two questions: Was the virtual assessment easy or hard for you to set-up? Did you feel comfortable performing the functional tests remotely?

### DEMOGRAPHIC AND CLINICAL DATA

Demographics (date of birth, sex, gender, and postal code of residence) were collected from the participants' medical records. Postal code was used to determine the geographical distribution of the participants (urban versus rural), where urban areas were defined as areas with a population of at least 1,000 and a density of 400 or more people per square kilometer (Singh, 2001).

### STATISTICAL ANALYSIS

Data were described using medians and interquartile ranges (25^th^ and 75^th^ percentiles). Feasibility questions for virtual assessment and prevalence of physical frailty were described using frequencies and percentages. Statistical Product and Service Solutions (SPSS) version 27 was used for statistical analysis.

## RESULTS

### ATTENDANCE AND DEMOGRAPHIC INFORMATION

Among the 41 SOT recipients approached by the study coordinator, 36 (88%) agreed to perform the virtual assessment. Two participants didn't have access to a tablet, laptop, or smartphone, and three declined because they felt uncomfortable performing the virtual assessment. Another two people did not answer the video-call on the day of their assessment. Of the seven individuals who didn't perform the virtual assessment, 2 (29%) resided in rural areas. A total of 34 (83%) participants completed the virtual assessment for physical frailty. The sample characteristics are shown in Table 1. The participants' median age was 62 (46-67) years of age, BMI = 25.7 kg/m^2^ (22.9-29.9), 15 (48%) were female, and lung transplant recipients were the predominant organ group represented in the sample (n = 26, 76%). Additionally, thirteen (38%) of the sample lived in rural areas.

**Table 1:** Characteristics of the Study Participants (n = 34)

CHARACTERISTICS	MEDIAN (IQR) OR (%)
**AGE (YEARS)**	62 (46-67)
**SEX (% FEMALE)**	16 (47%)
**BMI (KG/M^2^)**	25.7 (22.9-29.9)
**RESIDENTIAL AREA**	
**URBAN**	21 (62%)
**RURAL**	13 (38%)
**TRANSPLANT TYPE**	
**HEART**	1 (3%)
**LIVER**	6 (18%)
**KIDNEY**	1 (3%)
**LUNG**	26 (76%)
**FUNCTIONAL MEASURES**	
**DASI (POINTS)**	42.7 (24.2-52.9)
**GAIT SPEED (M/S)**	1.2 (1.1-1.3)
**5 TIMES SIT TO STAND (SECONDS)**	7.7 (6.6-11.1)

*Note*. BMI: Body mass index. DASI: Duke Activity Status Index.

### FEASIBILITY OF THE VIRTUAL ASSESSMENT

The virtual assessment had a median session duration of 12 minutes (10-15 minutes). The reasons for assessments > 15 minutes (n = 8, 24%) were: issues with logging into the Microsoft Teams link (n = 4, 50%) or not having watched the video demonstration before the video call. A companion was present in 23 (68%) virtual assessments. All the participants could perform the tests independently without a gait aid or being physically assisted by the companion to complete any functional tests. The companion only helped with the camera positioning and with the video-call set-up. One participant (3%) did not have a 5-meter open space for the gait speed test, so this participant was instructed to set-up the alternative distance of 4 meters, which can also be used in the SPPB (Guralnik et al., 1994).

All participants indicated that the virtual assessment was easy to set-up, and they felt comfortable performing all tests. The internet connection was good, without gaps or speed delays in the assessment, and no adverse events (falls, dyspnea, or pain) were reported.

### MODIFIED PHYSICAL FRAILTY ASSESSMENT

**Figure 2:**
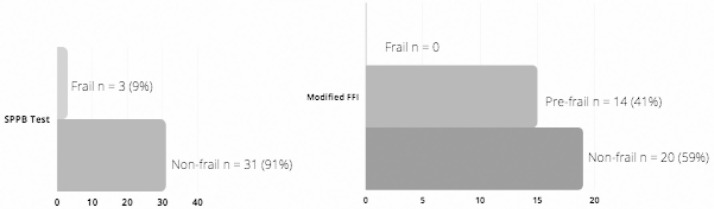
Classification of Frailty in SOT Recipients Using the Short Physical Performance Battery and Modified Fried Frailty Index

The mFFI and SPPB scores are shown in Figure 2. Exhaustion (n = 10, 39%) and weakness (n = 7, 21%) were the most prevalent FFI components, and slowness, low activity, and shrinking was present in only one participant each. The SPPB total score had a median of 12 (11-12). All participants were able to complete the SBBP test. Although the 5-times sit-to-stand test was the most challenging, 8 (24%) participants scored between 0-3/4 on this component. Static standing balance assessed by the SPPB had a ceiling effect, with all the participants scoring the maximum 4 points (ability to maintain all three tandem stance tests for 10 seconds each). Table 2 shows a cross-referenced analysis of frailty classification of the mFFI (columns) and SPPB (rows). Twenty (59%) of the participants were classified as non-frail by both frailty instruments. Three (9%) participants classified as frail by the SPPB were classified as pre-frail by the mFFI; and 11 (32%) of the participants characterized as pre-frail based on the mFFI were non-frail according to the SPPB. None of the participants were classified as frail for both instruments.

**Table 2:** Classifications of Frailty Cross-referenced between the SPPB and mFFI in SOT Recipients, 12 Months Post-transplant (n = 34)

		MODIFIED FFI		
		Frail	Pre-frail	Non-frail
**SPPB**	Frail	0	3 (9%)	0
	Non-Frail	0	11 (32%)	20 (59%)

*Note.* SPPB: Short Physical Performance Battery. FFI: Fried Frailty Index.

## DISCUSSION

Virtual assessment of physical frailty was feasible to perform in a cohort of SOT recipients with prior experience doing these tests in an in-person environment. The physical functional tests performed using the virtual assessment were safe and easy for the participants. The virtual assessment also has the potential to identify frail individuals at 12 months post-transplant. However, different instruments may not classify individuals along the frailty spectrum in the same way, which highlights the need to validate a frailty instrument in SOT recipients. Virtual assessments of physical frailty may be used in the future as part of the clinical practice to support the recent increase in telehealth delivery (Ju-Su et al., 2018).

Among the SOT recipients in this study, pre-frailty by the mFFI was present in 15 (44%) participants at 12 months post-transplant. This is similar in prevalence to the in-person assessment of physical frailty after lung, kidney, liver, or heart transplantation (Harhay et al., 2929; Jha et al., 2017; Lai et al., 2018; MacDonald, 2021; Varughese et al., 2020). The simple question about opening a jar may be an alternative to handgrip strength for evaluating muscle weakness remotely. This question has previously been used in a physical frailty model in a lung transplant candidate, where the physical frailty model that included this and other alternative methods to measure physical frailty had a modest agreement with the traditional Fried index (r = 0.38, 95% CI = 0.09-0.67) (Rozenberg et al., 2018). The same relationship between this question and the handgrip strength test was observed in older women (r = 0.49, p < 0.001), and a weak association was found in older men (r = 0.36, p = 0.015) (Simard et al., 2012). Another option for measuring grip strength at home, using less expensive equipment than a dynamometer, is a manual sphygmomanometer (Denby et al., 2013). This alternative method is reliable with the traditional Jamar handgrip dynamometer (rho = 0.75, p < 0.001), where the formula to convert sphygmomanometer scores to Jamar values is (Jamar = 0.54 × sphygmomanometer – 45.12) (Hamilton et al., 1992).

Compared with in-person assessment, virtual assessments require increased responsibilities for the participant and the participant's companion, including the set-up of the space for the video-call and familiarity with using video conferencing software. However, participants don't need to drive to the rehabilitation centers. In this study, the introductory phone call and the video demonstration sent before the video-call to explain the functional tests were essential components for the virtual assessment. These steps provided each participant with information about how the functional tests would be conducted and how they could prepare their space before the assessment, giving them the confidence to perform the physical tests independently in their home environment. We recommend implementing these steps to improve the feasibility of conducting functional tests remotely.

Our study reported no adverse events during the virtual assessment of physical function. Guidelines have been published to support the use of virtual assessments (Portnoy et al., 2020) as a safe alternative for in-person visits. These guidelines also state that adverse events are uncommon in virtual rehabilitation (Dechman et al., 2020). Similarly, another study that conducted a video-call for the 5-times sit-to-stand test in healthy older adults (Park et al., 2021) also reported no issues with safety.

In our study, the virtual assessment attendance was high, with 83% of those approached participating in the assessment. A companion was present in 68% of the video-calls, which may have also contributed to the success of the virtual assessment. Another Canadian study (Goodarzi et al., 2021) with 330 older adults observed that frail older adults without a companion usually have lower attendance in virtual assessments than phone call appointments. This may be due to inexperience with technology, which requires assistance from a companion. Our study also found that our participants did not need physical support to do the functional tests, but the companion often assisted with camera set-up. Many older people with and without chronic diseases live alone. So, more studies focusing on the feasibility of telehealth are needed for older people or people with mobility impairment living without a support person.

Beyond the familiarity with the internet environment, internet quality is essential for virtual assessment. In our study, the participants didn't have problems with an internet connection, even though 38% were from rural areas in Canada. The ability to do video calls with individuals in remote and northern regions and Indigenous communities can be limited, which can also further health inequity (Crawford & Serhal, 2020) in telehealth delivery, since telecommunications are not equally distributed across Canada (Ali-Hassan et al., 2020).

This study has limitations regarding the sample as it was from a single transplant center and had a significant higher proportion of lung transplant recipients than other organ types. We did not have participants using supplemental oxygen, with cognitive or mobility impairments, nor non-English speakers, so our results, particularly on feasibility, do not apply to individuals who may require more assistance from a companion. The balance component from the SPPB was not sensitive enough for the participants of this study, probably because they were already 12-months post-transplantation; other tests such as the Functional Reach Test (Duncan et al., 1999) or Berg Balance Scale (Berg et al., 1992) can be more accurate for this higher functioning population. However, the safety of these dynamic balance tests conducted virtually needs to be investigated.

## CONCLUSIONS

The virtual assessment of physical frailty in SOT recipients was feasible. It was short, with no adverse events, and participants felt confident performing it from home. This suggests that virtual assessments can be an alternative for assessing physical frailty in SOT recipients. Further studies should compare virtual and in-person assessments to determine the validity and reliability of virtual testing and include participants with lower levels of functioning. The predictive validity of frailty instruments also needs to be verified specifically in SOT recipients, to determine which instrument is most clinically relevant in this population.
